# Extensively Drug-resistant Tuberculosis, Italy and Germany

**DOI:** 10.3201/eid1305.060200

**Published:** 2007-05

**Authors:** Giovanni Battista Migliori, Johannes Ortmann, Enrico Girardi, Giorgio Besozzi, Christoph Lange, Daniela M. Cirillo, M. Ferrarese, Giuseppina De Iaco, Andrea Gori, Mario.C. Raviglione, SMIRA/TBNET Study Group

**Affiliations:** * Salvatore Maugeri Foundation, Tradate, Italy; † Bad Lippspringe Hospital, Germany; ‡ Ospedale Lazzaro Spallanzani, Rome, Italy; § Eugenio Morelli Hospital, Sondalo, Italy; ¶ Research Center Borstel, Borstel, Germany; # S. Raffaele Institute, Milan, Italy; ** Niguarda Hospital, Milan, Italy; †† University of Brescia, Brescia, Italy; ‡‡ University of Milan, Milan, Italy; §§ World Health Organization, Geneva, Switzerland

**Keywords:** Tuberculosis, drug resistance, multidrug-resistant tuberculosis, extensively drug-resistant tuberculosis, letter

**To the Editor:** Twenty-three countries have reported >1 case of extensively drug-resistant tuberculosis (XDR TB) (*1*); however, information about XDR TB is still incomplete. In particular, the response of XDR TB to treatment in countries with low incidence is not known. We compared mortality rates from XDR TB with those from multidrug-resistant (MDR) TB.

We analyzed data from all culture-confirmed TB cases diagnosed during 2003–2006 by the TB clinical reference centers in Italy (Sondalo, Milan, Rome) and Germany (Borstel, Grosshansdorf, Bad-Lippspringe) and reviewed original clinical records. Drug susceptibility testing for first- and second-line anti-TB drugs was performed according to World Health Organization (WHO) recommendations by quality-assured laboratories and retested at WHO Supranational Reference Laboratories (Rome/Milan; Borstel) (*2**–**4*).

XDR TB was defined as resistance to at least rifampin and isoniazid (MDR TB definition) in addition to any fluoroquinolone and >1 of 3 injectable anti-TB drugs (capreomycin, kanamycin, amikacin) (*3*). Characteristics of MDR TB and XDR TB cases were compared by χ^2^ test (categorical variables), Student *t* test (admission days), and Kaplan-Maier curve (sputum smear, culture conversion), where appropriate.

Of 2,888 culture-positive TB cases analyzed (Italy 2,140, Germany 748), 126 (4.4%) were MDR (Italy 83, Germany 43) and 11 (0.4%) were XDR (Italy 8, Germany 3). We estimate that the TB cases analyzed represent 24% of culture-positive cases reported in Italy (69.7% of MDR) and 4.2% of those reported in Germany (12.6% of MDR). XDR TB was diagnosed in each year of the study. All 11 XDR TB patients were receiving retreatment, and of the 126 MDR TB patients, 74 (58.7%) were receiving retreatment. All XDR TB patients were HIV seronegative; and of 109 MDR TB patients tested for HIV, 10 (9.2%) were HIV seropositive. Details about previous treatment regimens, drug resistance, and duration of treatment of XDR TB patients are summarized in the Appendix Table. XDR TB patients were significantly more likely than MDR TB patients to be resistant to all first-line drugs (8/11 vs. 36/126, p<0.005); 2 of these patients were resistant to all tested drugs (Appendix Table).

In Germany, nonnationals accounted for 95.3% (41/43) of MDR-TB cases and 100% (3 of 3) of XDR TB cases (all from the former Soviet Union); in Italy, they accounted for 72.3% (60/83) and 50% (4/8), respectively (p<0.001). Of 126 patients with MDR, 8 (6.3%) died, 45 (35.7%) were treated successfully, 67 (53.2%) were still receiving treatment (after achieving bacteriologic conversion, radiologic and clinical improvement, or both) and 6 defaulted (4.8%). Of 11 patients with XDR, 4 (36.4%) died and 7 (63.6%) were still receiving treatment. Compared with MDR TB patients, XDR TB patients had a 5-fold higher risk for death (relative risk 5.45; 95% confidence interval 1.95–15.27; p<0.01) and required longer hospitalization (mean ± SD 241.2 ± 177.0 vs. 99.1 ± 85.9 days; p<0.001) and longer treatment durations (30.3 ± 29.4 vs. 15.0 ± 23.8 months; p<0.05). Smear and culture conversions were observed for 4 XDR TB patients compared with 102 MDR TB patients (smear median 110 vs. 41 days; culture median 97.5 vs. 58 days, respectively); time to smear and culture conversion significantly differed between the 2 groups (p<0.01). A higher percentage of XDR TB than MDR-TB patients had received previous anti-TB treatment (100% [11/11] vs. 59% [74/126], respectively, p<0.01) and were >45 years of age (64% [7/11] and 23% [29/126], respectively, p<0.01). Radiologic patterns of the thorax did not differ between XDR TB and MDR TB patients. In the overall sample, the only variable significantly associated with death (other than XDR TB status) was immigrant status (p<0.01). The association between XDR TB status and risk for death remained significant after stratification by immigrant status (p<0.05).

Our findings suggest that mismanagement of TB cases plays a major role in emergence of the problem in Europe (along with suboptimal infection control in congregate settings) (*5*), while in high HIV-prevalence settings (e.g., South Africa) XDR TB was mainly observed in patients never treated previously (*6*). Mortality rates among MDR TB patients treated in reference centers (6.3%) were lower than the rate observed in a previous study in general hospitals in Italy (8.7%) (*5*), although a proportion of our MDR TB patients are still completing treatment. This difference in rates is probably due to better management of MDR in the reference centers. Because of the high proportion of XDR TB patients still receiving treatment, further follow-up is necessary to assess potential for cure. The clinical relevance of resistance to all first-line drugs or other factors (e.g., delayed or inadequate treatment, suboptimal observation of drug intake) as major determinants of death needs further evaluation. The appearance of XDR TB in western Europe confirms that poor management and poor infection control in congregate settings exist and that new rapid diagnostic tests and new drugs are urgently needed.

## Supplementary Material

Appendix Table XDR TB patient characteristics, Italy and Germany, 2003-2006*

## References

[1] Migliori GB, Loddenkemper R, Blasi F, Raviglione MC. 125 years after Robert Koch’s discovery of the tubercle bacillus: the new XDR-TB threat. Is “science” enough to tackle the epidemic? Eur Respir J. 2007;29:423–7. 10.1183/09031936.00001307 17329486

[2] Laszlo A, Rahman M, Espinal M, Raviglione M; WHO/IUATLD Network of Supranational Reference Laboratories. Quality assurance programme for drug susceptibility testing of *Mycobacterium tuberculosis* in the WHO/IUATLD Supranational Laboratory Network: five rounds of proficiency testing 1994–1998. Int J Tuberc Lung Dis. 2002;6:748–56. 12234129

[3] World Health Organization. Extensively drug-resistant tuberculosis (XDR-TB): recommendations for prevention and control. Wkly Epidemiol Rec. 2006;81:430–2. 17096498

[4] Shah NS, Wright A, Bai G-H, Barrera L, Boulahbal F, Martín-Casabona N, Worldwide emergence of extensively drug-resistant tuberculosis. Emerg Infect Dis. 2007;13:380–7. 17552090 10.3201/eid1303.061400 PMC2725916

[5] Ferrara G, Richeldi L, Bugiani M, Cirillo D, Besozzi G, Nutini S, et al. Management of multidrug-resistant tuberculosis in Italy. Int J Tuberc Lung Dis. 2005;9:507–13. 15875921

[6] Gandhi NR, Moll A, Sturm AW, Pawinski R, Govender T, Lalloo U, et al. Extensively drug-resistant tuberculosis as a cause of death in patients co-infected with tuberculosis and HIV in a rural area of South Africa. Lancet. 2006;368:1575–80. 10.1016/S0140-6736(06)69573-1 17084757

