# Supplementary material for: Extensively Drug-resistant Tuberculosis, Italy and Germany
Source: Emerg Infect Dis. 2007 May;13(5):780–2. doi: 10.3201/eid1305.060200 (PMC2738462; doi:10.3201/eid1305.060200)
Supplement: Appendix Table — XDR TB patient characteristics, Italy and Germany, 2003-2006* [file 007-0200_appT-s1.pdf]

Appendix Table. XDR TB patient characteristics, Italy and Germany, 2003–2006*

Age (y), sex	Country: Of diagnosis	Country: Of birth	Radiologic cavities, thorax†	No. treatment periods‡	Drugs received§	Drug resistance†§	Hospitalization, d	Smear conversion, d	Culture conversion, d	Treatment duration, mo	Outcome¶
40, M	Italy	Italy	Bilateral	3	S, R, H, E, Z; Rb	S, R, H, E, Z; FQ, AK, Cyc	220	–	–	73	Improved
61, F	Italy	Peru	Monolateral	1	S, R, H, E, Z; FQ, Eth, Ak, PAS, Cyc	S, R, H, E, Z; FQ, AK, Cyc	83	–	–	16	Died
27, F	Italy	Moldova	Monolateral	3	S, R, H, E, Z; FQ Eth AK, PAS, K, Cyc, Clof	S, R, H, Z; FQ, Eth, K	406	300	300	10	Improved
46, M	Italy	Senegal	Bilateral	4	S, R, H, E, Z; FQ, PAS, Cyc	S, R, H, Z; FQ, Eth, AK, PAS	69	49	35	6	Improved
43, F	Italy	Italy	Bilateral	3	S, R, H, E, Z; FQ, Eth, AK, PAS, C, K, Cyc, Rb, Clof, Dap, Cl, Th	S, R, H, E, Z; FQ, Eth, AK, PAS, C, K, Cyc, Rb, Clof	422	–	–	94	Died
38, F	Italy	Italy	Bilateral	3	S, R, H, E, Z; FQ, Eth, PAS, C, Cyc	S, R, H, E, Z; FQ, AK, PAS, Cyc	80	–	–	12	Died
49, F	Italy	Italy	Bilateral	3	S, R, H, E, Z; FQ, Eth, AK, PAS, C, K, Cyc, Rb, Clof, Dap, Cl, Th	S, R, H, E, Z; FQ, Eth, AK, PAS, C, K, Cyc, Rb, Clof, Dap, Cl, Th	625	–	–	60	Died
33, M	Germany	Uzbekistan	Monolateral	2	S, R, H, E, Z	S, R, H, E, Z; FQ, Eth, AK, C, Rb	120	–	–	4	Improved
29, M	Germany	Uzbekistan	Bilateral	2	S, R, H, E, Z; Rb	S, R, H, E, Z; FQ, C	240	180	160	12	Improved
52, M	Germany	Azerbaijan	Monolateral	>1	Not available	S, R, H, E, Z; FQ, AK, PAS, C, Cyc, Rb	140	7	35	6	Improved
36, F	Italy	Romania	Bilateral	4	S, R, H, E, Z; FQ, Eth, PAS, Cyc, Rb	R, H, Z; FQ, AK, Rb	248	110	–	28	Improved

*XDR TB, extensively drug-resistant tuberculosis; –, not achieved. All patients were HIV seronegative.
†At time of XDR TB diagnosis.
‡Treatment periods >30 d.
§First-line drugs: S, streptomycin; R, rifampin: H, isoniazid, E, ethambutol; Z, pyrazinamide; second-line drugs: FQ, fluoroquinolone; Eth, ethionamide; AK, amikacin; PAS, para-aminosalycilic acid;

C, capreomycin, K, kanamycin; Cyc, cycloserine; other drugs: Rb, rifabutin; Clof, clofazimine; Dap, dapsone; Cl, clarithromycin; Th, thiacetazone.
¶Improved, achieved bacteriologic conversion, radiologic and clinical improvement, or both.
